# Assessment of pollution and risks associated with microplastics in the riverine sediments of the Western Ghats: a heritage site in southern India

**DOI:** 10.1007/s11356-022-24437-z

**Published:** 2022-12-03

**Authors:** Kaniyambadi Amrutha, Sachin Shajikumar, Anish Kumar Warrier, Joju George Sebastian, Yamuna Adichinalniravel Sali, Thara Chandran, Sanitha Sivadas, Ravidas Naik, Vadakkeveedu Narayan Amrish, Arun Kumar, Vishnu Unnikrishnan

**Affiliations:** 1 Department of Civil Engineering, Manipal Institute of Technology, Manipal Academy of Higher Education, Manipal, 576104 Karnataka India; 2 Centre for Climate Studies, Manipal Academy of Higher Education, Manipal, 576104 Karnataka India; 3 Nitte (Deemed to Be University), Department of Public Health Dentistry, AB Shetty Memorial Institute of Dental Sciences (ABSMIDS), Mangalore, 574199 Karnataka India; 4 National Centre for Coastal Research, NIOT Campus, Velacherry-Tambaram Main Road, Pallikaranai, Chennai - 600100 India; 5 National Centre for Polar and Ocean Research, Headland Sada, Vasco-da-Gama, 403804 Goa India

**Keywords:** Microplastic pollution, Rainfall, *Pristine* river, Heritage sites, Western Ghats, Southern India

## Abstract

There is very little knowledge on microplastic pollution in the Western Ghats (WG), a heritage site in southwest India. To address this, we have studied the spatiotemporal variations of sedimentary microplastics (MPs) from the River Sharavathi, *a pristine river* in the Western Ghats (WG), southern India. The rich biodiversity in the region makes it relevant to analyse the distribution of this emerging pollutant that is causing harm to the biota and the ecosystem. We analysed the sedimentological and carbon content (organic and inorganic) of these sediments and explored their relationship with MPs. Finally, risk assessment indices such as the Pollution Load Index (PLI), the Polymer Hazard Index (PHI), and the Potential Ecological Risk Index (PERI) were calculated to detect the levels of plastic pollution. The concentration of MPs ranged from 2.5 to 57.5 pieces/kg and 0 to 15 pieces/kg during the pre-monsoon and post-monsoon seasons, respectively. The dip in the MPs’ abundance during the post-monsoon season was due to the extremely high rainfall in the river basin during July–August 2019, which would have entrained the sedimentary MPs and transported them to the coast/Arabian Sea. Smaller MPs (0.3–1 mm) were more abundant than the larger MPs (1–5 mm), mainly due to the breakdown of sedimentary plastics by physical processes. Fragments, films, foams, and fibres were the main categories of MPs, and the main polymers were polyethylene, polyethylene terephthalate, and polypropylene. No significant relationship was observed between the sedimentological properties and microplastics, which may be due to the different physical properties of sediments and microplastics. The PLI, PHI, and PERI indices suggest different contamination levels in the river basin. Based on the PLI scores, all the samples belong to the hazardous level I suggesting minor risk category, and the risk of microplastic pollution falls under the high to hazardous risk category based on the PHI values. The PERI value ranged from 160 to 440 and 40 to 2240 during the pre-monsoon and post-monsoon seasons, respectively. The risk assessment in a region known for its rich biodiversity is crucial, as the data can be used by the district administration to mitigate plastic pollution.

**Supplementary Information:**

The online version contains supplementary material available at 10.1007/s11356-022-24437-z.

## Introduction

Microplastics (MPs) are tiny (< 5 mm) particles of plastic debris (Thompson et al. 2004). The chemical properties of plastic materials help them persist in nature for a long time and disintegrate into MPs (Browne et al. 2007) due to biotic and abiotic processes (Ji et al. 2021). Micro- and nano-sized plastic materials are known to adsorb harmful chemical pollutants (Hahladakis et al. 2018) from water bodies due to their longer residence times (GESAMP 2015). As a result, these small, polluted plastic materials get easily ingested by all organisms belonging to the marine food chain (Rochman et al. 2013). Consumption of marine organisms can also lead to the transfer of MPs to the human body (Sharma and Chatterjee 2017), affecting their health. Besides the food chain, MPs can also reach the human body through other pathways, including atmospheric transmission (Wang et al. 2021).

Rivers play a significant role in transporting plastic debris of different sizes into the marine system (Ockelford et al. 2020). According to a global estimate of plastic pollution, riverine MPs that enter the marine environment exceed 2 million tonne per year (Lebreton et al. 2017). According to Meijer et al. (2021), over a thousand rivers contribute about 0.8–2.7 million metric tonnes of plastic waste per year, or about 80% of global annual emissions. With the help of hydrodynamic modelling involving sediment transport, He et al. (2021) concluded that river sediments are likely to be a sink for MPs rather than act as a carrier into the oceans. Highly dense MPs can quickly sink through the water column and mix with the sediments (Waldschläger et al. 2022). whereas less dense MPs can get deposited after forming biofilms on their surface, increasing their density (Crawford and Quinn 2017). Hydrodynamic conditions mainly influence the transport mechanisms of MPs in sediments in the river channel, along with the physical properties of the plastic materials and sediments (Yang et al. 2021). These factors affect the movement of microplastic particles and determine their retention period in the sediments. Some authors pointed out that the behaviour of small plastic materials can be similar to sediment particles with hydraulically identical physical properties (Harris 2020; Ockelford et al. 2020). Gerolin et al. (2020) reported the pervasive occurrence of MPs in the sediments of large Amazon rivers. They attributed it to the collective influence of anthropogenic activities and hydraulic conditions in the rivers. A greater abundance of MPs can affect the physical properties of sediments and soils, such as porosity (Adomat and Grischek 2021), permeability (Carson et al. 2011), and bulk density (de Souza Machado et al. 2018). Furthermore, the deposited MPs can re-enter the riverbed due to increased turbulent energy at the water–sediment boundary or bioturbation activity (Ockelford et al. 2020; Ji et al. 2021). Besides, high rainfall events in the riverine catchment may induce floods, which can play a significant role in flushing out the MPs from continental sediments into the marine environment (Hurley et al. 2018).

Microplastics research in India has skewed chiefly towards the coasts (Veerasingam et al. 2020; Amrutha et al. 2021), and terrestrial water bodies have received less attention. However, a few studies on microplastic pollution in riverine sediments from Indian rivers exist (Sarkar et al. 2019; Amrutha and Warrier 2020; Patel et al. 2020; Ram and Kumar 2020; Chauhan et al. 2021; Singh et al. 2021; and Tsering et al. 2021, Table 1). Besides, there is little knowledge on the behaviour of MPs with sedimentary and organic properties in these river systems. Addressing this issue is essential as river systems in India experience high rainfall during the southwest monsoon season, leading to floods in the river catchment. Excessive flooding can transfer many materials (including plastics of different sizes) into the Arabian Sea. The impact of microplastic pollution is widespread and has threatened even heritage sites (Kutralam-Muniasamy et al. 2021; Khaleel et al. 2022). For example, the water and sediment samples from the Hashilan Wetland (a national heritage site in northwestern Iran) contain many MPs (Abbasi 2021), mainly concentrated near roads, agricultural farms, and tourist places. The beach sediments of Cartagena (a world heritage site in the Colombian Caribbean) were found to be heavily polluted with microplastics and showed higher levels of trace elemental concentrations (Acosta-Coley et al. 2019).

**Table 1:** A summary of the research on microplastics from riverine sediments in India

Sl. no	River name	No. of samples	Sampling tool	Salt solution used for density separation	Average (± SD) MP abundance	Units	MP size	MP categories	Instrumentation	Polymer composition	References
1	Ganga	07	Stainless steel spoon	Zinc chloride	37.56 ± 16.5	ng/g	63–850 µm and 850 µm–5 mm	Fibre, filament, film, foam, bead, fragmented plastic	Stereomicroscope (Nikon) and ATR-FTIR	PET, PE, PP, PS	Sarkar et al. (2019)
2	Netravathi	10	Stainless steel spoon	Zinc chloride	96 ± 82.86	Pieces/kg	0.3–1 mm and 1–5 mm	Fibre, foam, film, fragment, pellet	Stereomicroscope (Nikon) and ATR-FTIR	PE, PET, PP	Amrutha and Warrier (2020)
3	Sabarmati	04	Stainless steel scoop	Sodium chloride	47.1 (large size) and 4 mg (small size) *	mg	76–212 µm and 212 µm–4 mm	Not studied	Not studied	Not studied	Ram and Kumar (2020)
4	Sabarmati	04	Stainless steel scoop	Sodium chloride	134.53 to 581.70*	mg/kg	76–212 µm and 212 µm–4 mm	Plastic debris, fibre	SEM–EDS	Not studied	Patel et al. (2020)
5	Ganga	05	Stainless steel scoop	Sodium chloride	25 35	Items/kg mg/kg	1 mm, 1–2.5 mm, 2.5–5 mm, > 5 mm	Fragment, foam, filament, film	Stereomicroscope (Leica) and ATR-FTIR	PES, PVC, PBAN, PVC/PE, PVT/B, PE-PP, PAI/PTM, PP, CL, PS, PE	Singh et al. (2021)
6	Alaknanda	04	Grab sampler	Sodium chloride	389*	Particles	< 1 mm, 1–2, 2–3, 3–4, 4–5 mm	Fragment, fibre, pellet, film	Stereomicroscope, SEM–EDS	Not studied	Chauhan et al. 2021
7	Brahmaputra	10	Stainless steel spoon	Sodium tungstate dihydrate	20–240* 531–3485*	MP/kg	20–150 µm 150–5000 µm	Fragment, fibre, bead	µ-FTIR	PE, PP, PA, Polyester, PTFE, PVC	Tsering et al. (2021)
8	Indus	08	Stainless steel spoon	Sodium tungstate dihydrate	60–340* 525–1752*	MP/kg	20–150 µm 150–5000 µm	Fragment, fibre, bead	µ-FTIR	PE, PP, PA, PES, PS	Tsering et al. (2021)
9	Sharavathi	10	Stainless steel spoon	Zinc chloride	14.82 ± 13.10 (PRM) 6.39 ± 5.17 (POM)	Pieces/kg	0.3–1 mm and 1–5 mm	Fibre, foam, film, fragment, pellet	Stereomicroscope (Nikon) and ATR-FTIR	PE, PET, PP	This study

^*^Average values not reported; *PRM*, pre-monsoon (April 2019); *POM*, post-monsoon (January 2020).

Therefore, this study aimed to quantify and characterise the spatiotemporal distribution of microplastics in the sediment samples of the River Sharavathi, a pristine waterway originating in the Western Ghats, one of the world’s heritage sites. The Western Ghats (WG) is one of the essential physiographic features in southwest India. It is a 1600 km long linear mountain chain with dense forests and an aerial coverage of more than 140,000 sq. km (Mudbhatkal and Amai 2018). Due to its dense vegetation, the WG plays host to several exotic species of flora and fauna (Ghate et al. 2004; Chandran et al. 2010). The rivers originating in the highland regions of the Western Ghats are an essential source of fresh water for the population living on the Western Ghats’ windward side, which receives copious rainfall during the southwest monsoon season (June to September) every year. The WG is one of the 35 hotspots of biodiversity recognised by UNESCO due to its richness and finds itself on the World Heritage List (Ramachandra et al. 2018). According to the IUCN’s Red Data List, more than 300 globally threatened species are found in the WG, and they are classified as vulnerable, endangered, and critically endangered. Recent studies have shown that the ecosystem of the WG is undergoing a drastic change due to rampant anthropogenic activities in the region (Bhat et al. 2014; Boominathan et al. 2014). The Sharavathi river basin is known for its rich biodiversity, encompassing various species of flora and fauna, including endemic and endangered species. Therefore, microplastic investigation in the sediments of this river is accompanied by risk assessment. This will help in understanding the threat they pose to organisms thriving in such pristine ecosystems. In addition, we have measured the sedimentary properties (sand, silt, clay, total organic carbon, and total inorganic carbon) and discussed their relationship with microplastics data.

## Materials and methods

### Study area

**Fig. 1:**
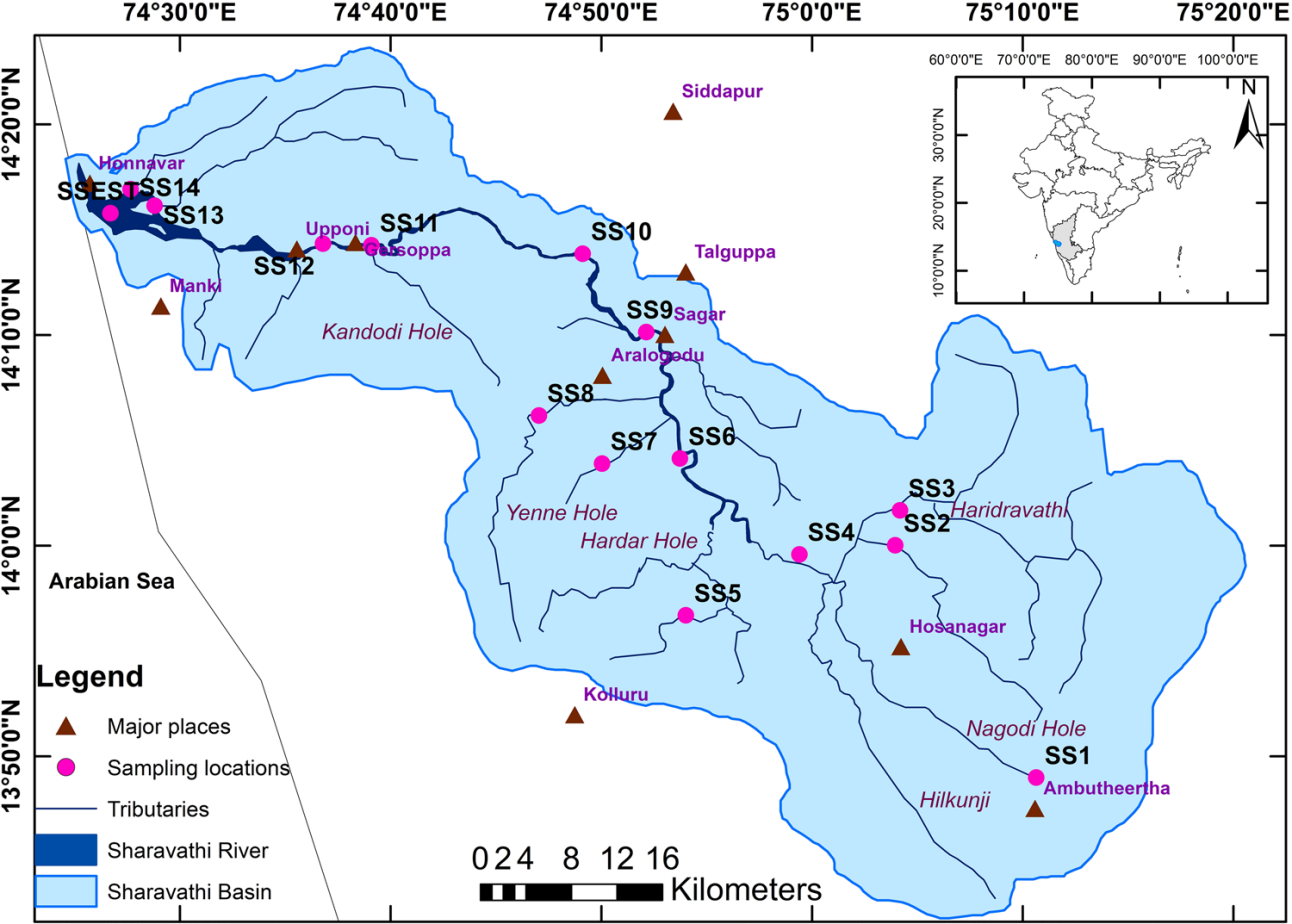
River Sharavathi basin with the sampling locations

The River Sharavathi originates at Ambutheertha, in the Theerthahalli Taluk of Karnataka, India (Fig. 1). The river basin falls under two districts of Karnataka, namely Shimoga and Uttara (North) Kannada, and the river travels nearly 128 km before meeting the Arabian Sea at Honnavar. This river basin covers an area of approximately 2784 km^2^. The river flows mainly through the forest area of the Western Ghats (*Sahyadris*) in the upstream region. The downstream part of the river is not affected much by anthropogenic activities compared to the other west-flowing rivers of Karnataka. However, places of importance, such as the Jog Falls and the Linganamakki Reservoir, are located on the river’s profile. Located in the Western Ghats, the river catchment receives copious rainfall between June and September (southwest monsoon); the annual mean precipitation in the basin ranges from 1200 to 5000 mm (Karthick and Ramachandra 2006). The river is recognised as a hot speck in the biodiversity hotspot, and the presence of many endangered ecosystems and life forms in the basin makes the river ecologically significant (Ramachandra et al. 2012).

### Sample collection and extraction of microplastics

Surface sediments (0–2 cm depth) were collected from the shorelines of the River Sharavathi and some of its tributaries during the pre-monsoon (PRM; *n* = 14; April 2019) and post-monsoon (POM; *n* = 9; January 2020) seasons. The 14 sampling sites were selected during PRM in such a manner that the entire river catchment was covered. During POM, due to the effects of monsoon 2019, some of the sites were not accessible, so we could collect only nine samples. Replicate sediment samples (from an approximate distance of 2 m) from each of the 14 and 9 stations were collected using a stainless steel spoon and carefully homogenised and packed in an aluminium container. The average weight of the sediments accumulated was around 1.5 kg, covering an area of 15–20 cm^2^. The samples were safely transported to the laboratory. Microplastics were extracted from the sediments using a modified version of the National Oceanic and Atmospheric Administration (NOAA) protocol (Masura et al. 2015). Around 400 g of the wet sediment sample was transferred into a 1-L beaker and oven-dried at 90 °C overnight (Masura et al. 2015; Rodrigues et al. 2018). The dry weight of the sediments was noted and later treated with 300 mL of sodium hexametaphosphate solution (5.5 g/L; Amrutha and Warrier 2020). The beakers were kept on the rotary shaker for an hour at 250 rpm to disaggregate the samples. The samples were wet-sieved by passing them through sieves (Haver standard test sieve) of 5 mm and 0.3 mm mesh sizes using double-distilled water (Masura et al. 2015). The plastic materials observed in the 5-mm sieve were kept separately. The particles in the 0.3-mm sieve were transferred to a 1-L beaker, and 300 mL of zinc chloride solution (933.3 g/L) was added for primary density separation (Rodrigues et al., 2018). Later, the floating particles were sieved through a 0.3-mm mesh and then transferred to a 500-mL beaker and oven-dried at 75 °C for 24 h. Organic matter in the sediments was digested using the wet peroxide oxidation (WPO) method (Masura et al. 2015). In this technique, 20 mL of a 0.05 M Fe(II) solution was added to the sample along with 20 mL of hydrogen peroxide (H_2_O_2_; 30%). The process was continued until all the organic matter was digested. The remaining particles were again treated with 300 mL of zinc chloride solution for the secondary density separation and kept overnight. The floating particles were passed through 1 mm and 0.3 mm sieves the next day.

### Visual identification of microplastics and polymer composition

The particles in the two sieves were transferred into different watch glasses, dried, and visually identified with the help of a stereo zoom microscope (Nikon) at a magnification of 40 × . During visual identification, we followed the visual criteria methods mentioned in Hidalgo-Ruz et al. (2012) to ensure we did not pick up non-plastic materials. The particles with no distinguishing features of plastic were not treated as plastic. Moreover, the suspected particles with unclear features were tested for chemical composition along with frequently occurring MPs. The recovered MPs were transferred into pre-weighed glass vials and then weighed. The MPs were morphologically classified into fibres, fragments, films, foams, and pellets. To determine the chemical composition of the polymers, frequently occurring and suspected MPs (PRM = 24, POM = 8) were analysed with the help of a Fourier transform infrared (FTIR) spectrometer affixed with an attenuated total reflectance (ATR) unit (Sarkar et al. 2019). Around 65% of the MPs used for ATR-FTIR analysis were in the 1–5 mm size range, and 35% were 0.3–1 mm. The frequently occurring MPs were classified into different groups based on their similarities, and proportionate samples from each group were subjected to FTIR analysis. The identification of the polymer composition of MPs was made with the help of Shimadzu IRSpirit FTIR with a QATR-S Single Reflection ATR Accessory. Percent transmission was recorded at a range of 3500–500 cm^−1^ with a resolution of 4 cm^−1^. Around twenty-five scans were performed for each sample. Chloroform was used to clean the ATR crystal each time before scanning. The chemical composition of polymer particles was identified by comparing the data with reference spectra (Xu et al. 2019). Open Specy (Cowger et al. 2020), an open-source spectra identification tool, was used to recognise the IR spectra (Pearson’s correlation coefficient > 0.8).

### Particle size analysis

Particle size analysis was performed using pipette analysis (Carver 1971). Each sediment sample (5 g) was treated with 20 mL of 30% H_2_O_2_, followed by 15 mL of glacial acetic acid (10%) to remove the organic and carbonate contents, respectively. After adding 15 mL of sodium hexametaphosphate (Calgon) solution, the sample was wet-sieved to separate the sand fraction (> 63 microns) using an ASTM mesh number 230. The silt and clay fraction (< 63 microns) was transferred into a 1000-mL measuring cylinder. The silt and clay fractions were pipetted from depths of 10 and 5 cm, respectively, at different time intervals according to Stokes’ law. The three fractions were oven-dried, and their percentages were calculated using their dry weights.

### Estimation of total organic and inorganic carbon (TOC and TIC)

Total organic carbon (TOC) and total inorganic carbon (TIC) were calculated by the loss-on-ignition (LOI) method (Dean 1974). First, the samples were taken in pre-weighed ceramic crucibles and oven-dried for 24 h at 105 °C. Next, the samples were allowed to cool in a desiccator to room temperature. Later, the samples were heated in a muffle furnace to 550 °C and 950 °C for about four and 2 h, respectively. Following each step, the dry weights (DW_105_, DW_550_, and DW_950_) of the sediments were noted. The percentage of loss-on-ignition of sediment samples (initial weight IW) at 550 °C (LOI_550_) and 950 °C (LOI_950_) and TOC and TIC were obtained using the equations given by Dean (1974, 1999):

$${\mathrm{LOI}}_{550}=100\;({\mathrm{DW}}_{105}-{\mathrm{DW}}_{550})/\mathrm{IW}$$

$${\mathrm{LOI}}_{950}=100\;({\mathrm{DW}}_{550}-{\mathrm{DW}}_{950})/\mathrm{IW}$$

$$\mathrm{TOC}={\mathrm{LOI}}_{550/2};\;\mathrm{TIC}={0.273\mathrm{LOI}}_{950}$$

### Data analysis

Statistical analysis of the entire dataset was carried out on SPSS 23.0 software (SPSS Inc., Chicago, IL, USA). The difference in various parameters between the two seasons was analysed using an independent *t* test. In addition, Pearson’s correlation coefficient analysis was performed in Microsoft Excel 2016 to check for any correlation between the properties of MPs and organic and inorganic carbon concentrations. A significance value of *p* < 0.05 was considered statistically significant. The remaining figures were drawn in multiple items of graphical software (Sigmaplot version 12.0, Microsoft Excel 2016, and ArcMap version 10.3).

### Risk assessment analysis

Pollution Load Index (PLI), Polymer Hazard Index (PHI), and Potential Ecological Risk Index (PERI) are a few indices that can be used to study the degree of microplastic contamination in the ecosystem and to evaluate their environmental harm to flora and fauna. According to Tomlinson et al. (1980), the PLI is used to assess the degree of environmental pollution. This study used PLI to gain information on the intensity of microplastic pollution in the sediments of the River Sharavathi. The PLI value at each sampling station is related to the microplastic pieces’ concentration factors (CF_*i*_). The equation used to derive PLI scores was:

$${\mathrm{CF}}_{\mathrm{i}}={C}_{\mathrm{i}}/{C}_{\mathrm{o}}$$

$$\mathrm{PLI}=\surd {CF}_{i}$$

CF_i_ is the ratio of the MP concentration at each sampling station (*C*_i_) to the background values of the MP concentration (*C*_o_). The lowest value obtained from the sediments collected during the pre-monsoon from a sampling site closer to the source region is considered the background value.

The index PHI is the product of the percentage of specific polymer types obtained at each sampling site (*P*_*n*_) and the hazard scores of polymer types (*S*_*n*_; Lithner et al. 2011; Ranjani et al. 2021). The total risk of microplastic pollution in the study area can be categorised into different hazard levels depending on the PHI scores. The formula PHI = Σ*P*_*n*_ × *S*_*n*_ was used to calculate the PHI of microplastics present in the sediments of the River Sharavathi. Another important indicator of ecological risk is the Potential Ecological Risk Index (PERI), which is used to evaluate the environmental impact of various pollutants in different environments (Peng et al. 2018). The following equations were used to derive the PERI scores:

$${C}_{f}^{i}= \frac{{C}^{i}}{{C}_{n}^{i}}$$

$${T}_{r}^{i}= \sum_{n=1}^{n}\frac{{P}_{n}}{{C}^{i}}*{S}_{n}$$

$${E}_{r}^{i} = {T}_{r}^{i} \times {C}_{f}^{i}$$

*C*^*i*^ is the concentration of contaminant ‘*i*’ (MP), and ${C}_{n}^{i}$ is the abundance of non-contaminated samples (= the least abundance of MPs found in the sediments). The coefficient of toxicity (${T}_{r}^{i}$) is indicative of the level of toxicity and biological sensitivity. The coefficient is the sum of the percentages of specific polymers in all the samples (*P*_*n*_/*C*^*i*^) multiplied by the hazard values of plastic polymers (*S*_*n*_) (Ranjani et al. 2021; Peng et al. 2018).

### Quality analysis and quality control

Standard protocols as outlined in the department laboratory were followed for this work. We adopted the necessary precautions to avoid contamination of the samples. No plastic accessories or items of equipment were used during sampling or processing. Only glass and steel apparatus were used, which helped control the quality. The laboratory where the samples were analysed was very clean, and the supply of outside air was minimal. Most of the processing has been done within the fume hood and laminar flow chamber. The workbench inside the laboratory was thoroughly cleaned with ethanol on all days. During the analysis, nitrile gloves and cotton aprons were worn. Milli-Q water was used to prepare solutions, and double-distilled water was used to clean all the lab apparatus and sieves. The sieves were pre-examined to ensure that no particles were attached to them. All the solutions were pre-filtered before analysis. Aluminium foil and watch glasses were used to cover the apparatus’s items to avoid contamination due to airborne particles. Two blank samples (double-distilled water) were also checked for the presence of microplastics. We could not collect blank samples in the field.

## Results and discussion

### Abundance, size, categories, and colour data of microplastics in the sediments of River Sharavathi

**Fig. 2:**
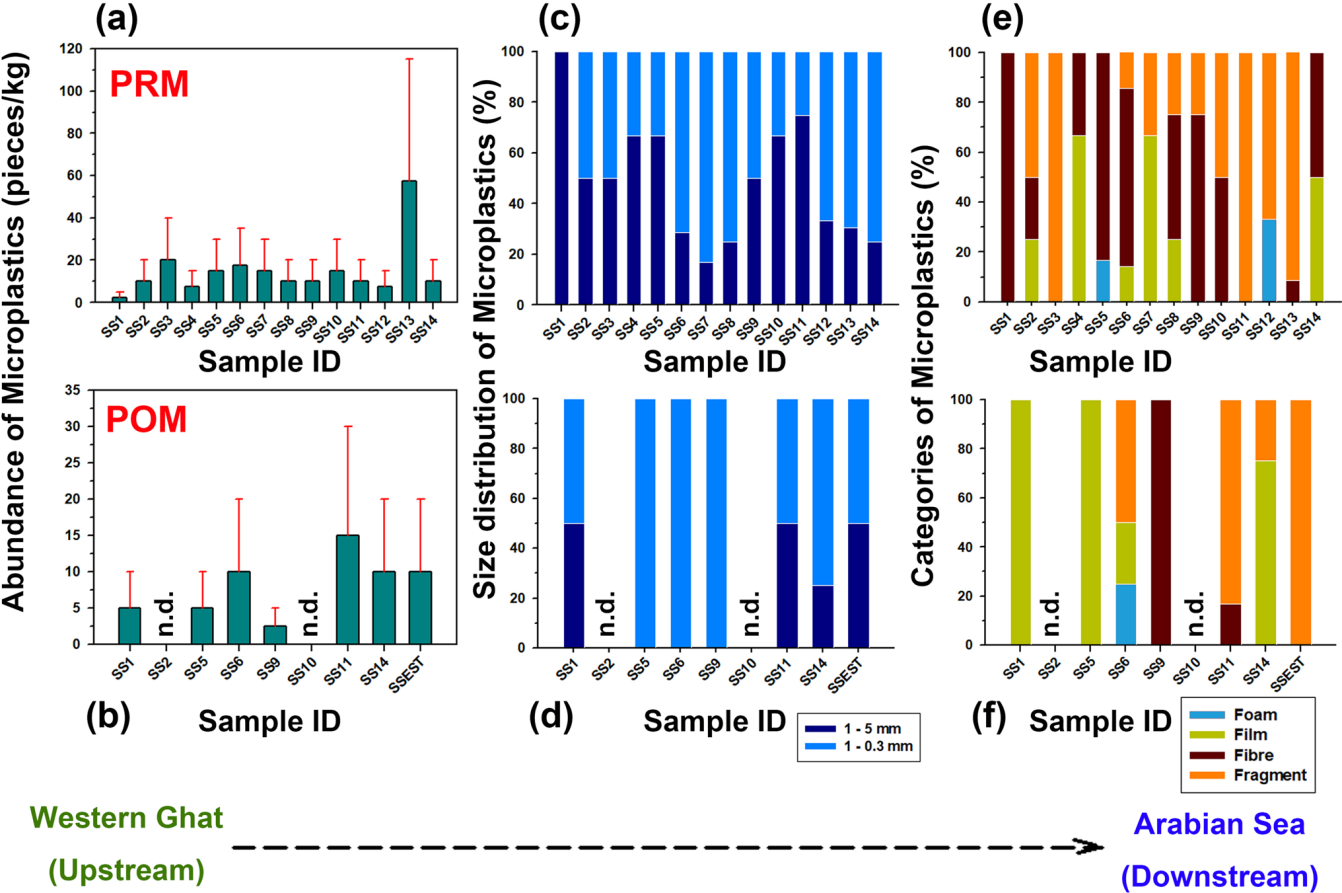
Microplastics data for sediment samples from the River Sharavathi and its tributaries collected during April 2019 (pre-monsoon) and January 2020 (post-monsoon) show the total abundance (**a** and **b**), percent abundance of the two size fractions (0.3–1 mm and 1–5 mm) (**c** and **d**), and percent abundance of different categories of microplastics (**e** and **f**), n.d. = not detected

The two procedural blanks (double-distilled water) showed the presence of four fibres in the size range of 0.3–1 mm. These have been corrected from the raw data. As the processing was carried out under the fume hood, the possibilities of airborne contamination were negligible. During the pre-monsoon, the station SS13 (Hosadmane) recorded the highest abundance of MPs (57.5 pieces/kg), and the station SS1 (Achakanya) showed a minor abundance (2.5 pieces/kg; Fig. 2a). Station SS1 is close to the source region of the river, and SS13 lies near the downstream section. The mean (± standard deviation) numerical abundance of MPs obtained from the catchment is 14.82 (± 13.10) pieces/kg. The higher quantity at SS13 can be attributed to the low flow rate (obstruction due to a small mid-channel bar) and increased residence time of MPs (Wang et al. 2021). Slower flow velocities allow the MPs to settle out of suspension and reach the sediment (Watkins et al. 2019). The presence of channel bars makes it a favourable site for the entrapment of MPs (Skalska et al. 2020), which explains the lesser quantity (10.0 pieces/kg) of MPs found at the following station, SS14, a location closer to the river mouth. The least abundance at SS1, the sampling site near the source, with a lower population (Population Census 2011), indicates a lesser intensity of anthropogenic activities in the region, which influences the distribution of MPs. The other locations with more MPs are stations SS3 (Tottadikoppe) and SS6 (Linganamakki). Wastewater and wastes entering the river catchment from the Haridravathi region due to agricultural activities (Rajinikanth and Ramachandra 2001) can be a reason for the relatively higher MPs at SS3. The microplastic pollution at station SS6 may be due to the ferry near the Linganamakki Reservoir. Furthermore, the sampling point SS6 is located upstream of the reservoir, where the abundance of MPs is generally higher than that in its downstream portion (Watkins et al. 2019). The locations SS5 (Tonikale) and SS7 (Valagere) have a higher population density (Population Census 2011) when compared to the other locations with less microplastic pollution; therefore, comparatively higher MPs were observed in these two stations (Liu et al. 2021). The location SS10 (near Jog Falls) is a tourist destination that attracts many visitors annually (Market Research Division, Department of Tourism, Government of India 2003). Tourists use the water of the Jog Falls for different activities like bathing and washing clothes, which can be an essential reason for the increased number of MPs in this region.

The post-monsoon data shows that the station SS11 (Gersoppa Ferry; 15 pieces/kg) showed a greater abundance of MPs (Fig. 2b). Apart from SS11, a relatively higher concentration of MPs was obtained from Linganamakki (SS6), Besinekere (SS14), and the estuary region (SSEST; Fig. 2b). The mean (± standard deviation) microplastic abundance is 6.39 (± 5.17) pieces/kg. No MPs were obtained in the sediments from stations SS2 (Sidyapura) and SS10 (Jog Falls; Fig. 2b). Statistically, there was no significant difference in the abundance of MPs between the two seasons. But, out of the eight samples collected during the two seasons, two sampling sites showed a slight increase in MP concentration during post-monsoon, while others showed a decrease in the microplastic abundance during post-monsoon (except Besinekere-SS14, where the values were the same for both the seasons). In general, the abundance of MPs was slightly lower during the post-monsoon compared to the pre-monsoon. One of the reasons for this is the heavy rainfall during July and August 2019, which might have entrained the MPs present in the sediments and flushed them to the coast of the Arabian Sea. With the decrease in water flow velocity during pre-monsoon, plastic particles may settle along with sediment particles (He et al. 2020). Apart from the higher number of MPs, the station SS11 showed a slight increase in their concentration compared to pre-monsoon. This results from factors like increased anthropogenic activities during the POM at the site. We did not observe a progressive increase or decrease of MPs from the source to the sink regions of the river catchment. The distribution of different-sized plastic materials in an area results from the combined effect of natural (hydrodynamic conditions, morphology, precipitation, sediment properties, and vegetation) and anthropogenic processes active in a river basin (Liu et al. 2021; Gerolin et al. 2020). The influence of tributaries in the transport of MPs into the main river can be another reason for the absence of a clear pattern (Kiss et al. 2021).

The percentage distribution of the two size ranges varied for each sampling site. The size distribution shows that 0.3–1 mm (57.83%) sized particles were higher than the 1–5 mm (42.17%) size range (Fig. 2c), which resonates with previous studies (Isobe et al. 2017; Amrutha and Warrier 2020). This is also the case in post-monsoon samples, where the smaller fraction (69.57%) is more abundant than the larger (30.43%; Fig. 2d). The MPs in the size range of 1 to 5 mm decreased significantly following the monsoon season of 2019. The smaller-sized particles pose a great danger to aquatic life because of their larger surface area, which helps them absorb harmful pollutants (Devriese et al. 2017). The difference in the minimum mesh size can result in a considerable difference in the estimation of microplastic concentration. In this study, we used a mesh size of 0.3 mm (Masura et al. 2015); a higher number of MPs might have been obtained if we had used sieves of less than 0.3 mm. Fragmentation of plastic materials deposited in the sediments by various physical actions, such as abrasion with coarser clastic materials, is essential for forming smaller MPs. The fragmentation rate mainly depends on several factors, such as the polymer type, residence time, and sediment grain size and shape (Chamas et al. 2020; van Wijnen et al. 2019).

**Fig. 3:**
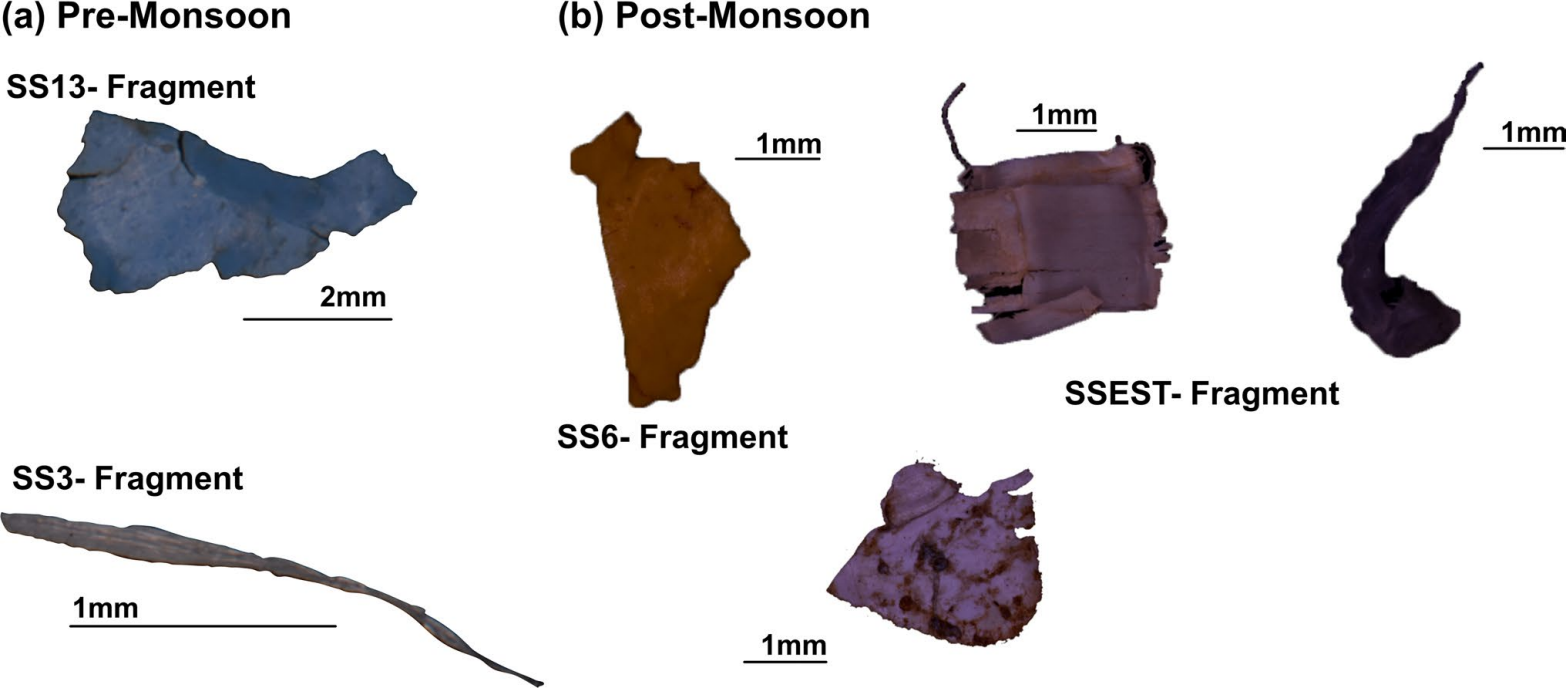
Microphotographs of the fragments found in this study. Scale bar is in millimetre

Categorization of the MPs showed that the fragments were the most abundant type (54.22%), followed by fibres (30.12%) and films (13.25%; Figs. 2e and 3). In the post-monsoon season, fragments (52.17%) predominated the samples, followed by films (34.78%) and fibres (8.70%; Fig. 2f). The turbulent conditions caused by the monsoon can result in the formation of smaller MPs, especially fibres suspended in the water column from the sediments and carried away (Bagaev et al. 2017). Foams were the least abundant category (PRM = 2.41%; POM = 4.35%), and pellets were not detected in any of the samples in both seasons (Fig. 2e and f). The fragments obtained from the samples are mainly derived from the degradation of plastic products like packing bags, including sacks, carry bags, and food containers (Zhang et al. 2015; Amrutha and Warrier 2020). The usage of sacks in constructing bunds across the river and in fencing was observed during the fieldwork. The fibres (Supplementary Fig. S1) are mainly attributed to synthetic textiles and garments; washing them will release many fibres (Browne et al. 2011) into the water bodies. In addition, ropes and fishing nets can also produce fibres (Andrady 2011). Films and foams are mainly broken pieces of polythene bags and packaging materials, formed as by-products of the fragmentation of these materials. Pellets mostly reach the rivers through cosmetics, personal care products like facial cleansers, and automobile industries (Mani et al. 2015), which were absent in the sediments. This implies the absence of these industries near the River Sharavathi and a smaller population that uses these commodities. A large number of secondary MPs show that mismanaged plastic waste is the main cause of pollution. This brings attention to the need for regulations and better management of plastic debris.

In the colour characterization, transparent (24.53%) and white (23.58%) MPs were the most abundant, followed by green (15.09%), brown (11.32%), and other coloured ones (< 10%; Supplementary Fig. S2). The primary sources of white and transparent MPs are mainly degraded carry bags made of plastic and other packing materials (Amrutha and Warrier 2020). Another source of these colours is that the weathering of MPs in the environment can lead to the loss of their original colour and cause them to become white or transparent (Xu et al. 2018). The coloured MPs are mainly the degraded parts of domestic plastic products like synthetic fabrics, packaging, and covers (Zhang et al. 2015; Wang et al. 2017). According to literature, white-coloured MPs are easily mistaken as prey by aquatic organisms and get ingested by them; also, green-coloured MPs, when entered into deep sea, will be mistaken as prey by organisms because of their bioluminescence (Crawford and Quinn 2017). The quantity of black-coloured particles decreased significantly post-monsoon.

Besides being a sink for MPs, rivers can carry these materials to the coastal environment and seas/oceans (Horton et al. 2017; Amrutha and Warrier 2020). Many studies have reported plastic pollution along the Karnataka coast, southwest India, including two beaches near the study area, Honnavar (Sridhar et al. 2009) and Kasarkod (Maharana et al. 2020). The results obtained in this study indicate that even though MPs were found in the sediments of the River Sharavathi, their quantity was less. The majority of the river flows through forest regions, and this is the main reason for the low level of microplastic pollution as plastic concentration decreases away from urban areas (Wang et al. 2017). According to Sridhar et al. (2009), most of the plastic debris accumulated in Honnavar is of local origin. In general, data on MPs from the river system in India is sparse. Only a few studies have reported the presence of microplastics in the riverine sediments (Table 1). Even though the sampling method, pre-treatment, and analysis vary among these studies, we have compared our data with other works using the same units of measurement adopted in this study. The numerical abundance obtained from the River Sharavathi during the pre-monsoon and post-monsoon seasons is 14.82 (± 13.10) pieces/kg and 6.39 (± 5.17) pieces/kg, respectively. The abundance is less than that of the rivers Netravathi (Amrutha and Warrier 2020), Ganges (Sarkar et al. 2019; Singh et al. 2021), Indus, and Brahmaputra (Tsering et al. 2021; Table 1). The difference in the concentration can be due to several reasons, including the sampling sites chosen, the seasons in which the samples were collected, and the size of the mesh used during sieving for extracting the MPs, population distribution, hydrodynamic parameters in the rivers, and improper disposal of plastic waste in the catchment.

### Polymer composition of microplastics

**Fig. 4:**
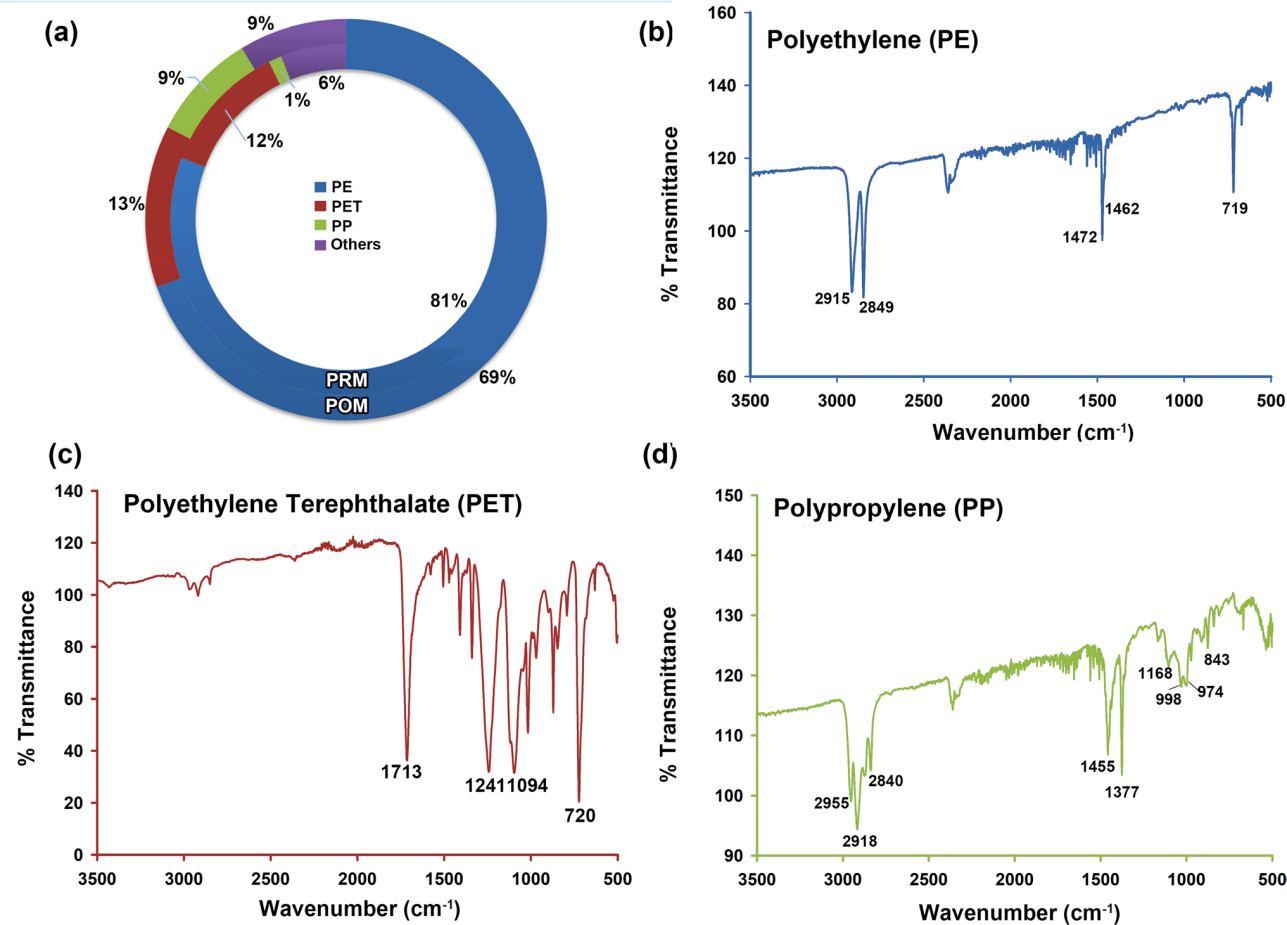
**a** A dough plot showing the abundance of major polymers found in this study. **b** to **d** FTIR-ATR spectral curves of selected microplastics from the River Sharavathi

The main polymers found in the sediment samples were polyethylene (PE; PRM = 80.72% and POM = 69.57%), polyethylene terephthalate (PET; PRM = 12.05% and POM = 13.04%), and polypropylene (PP; PRM = 1.20% and POM = 8.70%; Fig. 4). The polymer composition of a few microplastic particles was not identified (PRM = 5 and POM = 2). Only a minor amount of PP was obtained from the samples. Maharana et al. (2020) reported that PE and PP are the dominant polymer types present along the western coast of India. Polyethylene is a widely used polymer, mainly for packing purposes and covers. Hence, their presence is explainable. The presence of sack-derived fibres (found to be PE) derived from sacks used to construct bunds was observed in most samples. Polyethylene terephthalate is mainly derived from clothes, blankets, bottles, etc. (Coppock et al. 2017). The majority of the fibres obtained from samples are identified as PET. Generally, high-density polymers like PET sink and get deposited in sediments (Waldschläger et al. 2022). In contrast, polymers like PE and PP are less dense, due to which they float on the water’s surface or remain suspended in the upper portion of the water column (Yang et al. 2021). The presence of low-density polymers in the sediments can be due to the enhancement of their density by the accumulation or adsorption of pollutants or the growth of biofilms on microplastic surfaces (He et al. 2021).

### The role of 2019 rainfall in the distribution of microplastics

**Fig. 5:**
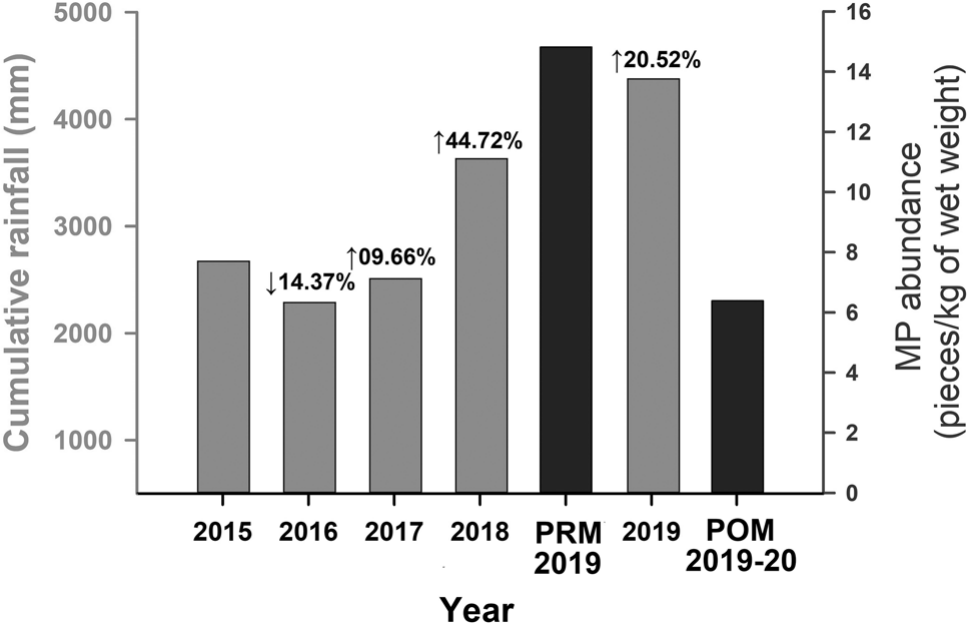
A bar diagram comparing the cumulative rainfall data received in the River Sharavathi basin between 2015 and 2019 (data obtained from Karnataka Power Corporation Limited; http://karnatakapower.com) and the abundance of microplastics obtained in the study during April 2019 (pre-monsoon) and January 2020 (post-monsoon). Compared to 2018, the basin got more than 20.5% more rain in 2019

In general, the abundance of MPs was slightly lower during the post-monsoon compared to the pre-monsoon. One of the reasons for this is that the regions (Uttara Kannada and Shivamogga) experienced extremely high southwest monsoonal rainfall during July–August 2019 compared to the average rainfall data (Fig. 5; Supplementary Figs. S3 and S4; Karnataka State Natural Disaster Monitoring Centre 2020). The cumulative rainfall record also substantiates this between 2015 and 2019 for the River Sharavathi basin. In 2019, the region received 20.5% more rainfall compared to the monsoon season of 2018 (Fig. 5; Karnataka Power Corporation Limited). Heavy rain can affect the distribution of MPs in the aquatic system. The increase in rainfall intensity can increase the erosion effect of surface runoff on land and, therefore, deliver plastic materials from the ground to water bodies, including rivers. Episodes of heavy rainfall, storm surge, or flood events can increase the plastic concentration mobilised by rivers at varying rates (Gündoğdu et al. 2018). Therefore, the intensity of the rainfall obtained in an area is an essential factor affecting the fate of MPs in the water and sediments in a river catchment. High flow velocity during the monsoon would have increased the entrainment capacity of the River Sharavathi, which mobilised the deposits (including MPs) on the riverbed. Remobilization results in more MPs being released from the sediments into the water column or surface water (Ockelford et al. 2020; Warrier et al. 2022). Hence, along with the microplastic input from terrestrial sources through surface runoff, the entrained MPs generated from the vertical exchange of sediments will be flushed out from the river to the sea or ocean due to excess rainfall. The increased flow rate and increased volume of water in the monsoon might have transported all these MPs from the river catchment to the Arabian Sea (Gündoğdu et al. 2018; Veerasingam et al. 2016). The residence times for MPs will be shorter due to the high energy conditions, making these materials flow towards the coast or sea (Hitchcock 2020). A recent study along the River Ganges stated that a higher amount of microplastic discharge to the Bay of Bengal occurred during the post-monsoon season (Napper et al. 2021).

### Relationship between sediment textural properties and characteristics of microplastics

Studying the particle size distribution and its relationship with MPs may help understand the influence of sediment processes on the abundance of MPs in the sedimentary environment. Besides microplastics, other organic and inorganic pollutants are known to vary with respect to sediment grain size (Sun et al. 2021). Particle size, TOC, and TIC data obtained for all the stations are shown in Supplementary Table S1. The dominant particle size found in the River Sharavathi sediments during the pre-monsoon season was sand (40.72% ± 24.36%), followed by clay (36.55% ± 19.31%), and silt (22.73% ± 10.59%). During the post-monsoon season, the sediment particle size was predominated by clay (42.26% ± 19.65%), whereas sand (29.57% ± 10.43%) and silt (28.17% ± 13.35%) showed near-similar distributions. The average TOC and TIC content during the pre-monsoon season were 0.64% ± 0.5% and 0.09% ± 0.04%, respectively. During the post-monsoon, the average values for the two variables decreased (TOC = 0.40% ± 0.18%, TIC = 0.07% ± 0.03%; Supplementary Table S1). The difference in the dataset between the two seasons is mainly attributed to the strong monsoonal rainfall in the river basin during July–August 2019 (Fig. 5 and Supplementary Figures S3 and S4), leading to an increase in the hydrodynamic conditions of the River Sharavathi and making it more turbulent.

Tables 2 and 3 show the relationship between microplastics’ characteristics and the particle size data of the River Sharavathi during the pre-monsoon (*n* = 14) and post-monsoon (*n* = 9). The abundance of MPs and sand content are negatively correlated (*r* =  − 0.26, *p* = 0.37, *n* = 14; Table 2) during the pre-monsoon season. The concentration of MPs exhibited an insignificant positive relationship with both silt (*r* = 0.40, *p* = 0.16), and clay fractions (*r* = 0.11, *p* = 0.71; Table 2). The abundance of microplastics showed an insignificant positive correlation with TOC during pre-monsoon (*r* = 0.20, *p* = 0.50; Table 2) and post-monsoon (*r* = 0.01, *p* = 0.98; Table 2). Microplastic abundance showed a positive (*r* = 0.36, *p* = 0.20) and negative (*r* =  − 0.03, *p* = 0.95) correlation with TIC before and after the monsoon, respectively (Tables 2 and 3). Previous studies have reported that the abundance of microplastics is negatively and positively correlated with sand and silt fractions, respectively (Goswami et al. 2021; Liu et al. 2021; Corcoran et al. 2020). A few studies have also documented a greater abundance of MPs in fine-grained sediments (Harris 2020). Interestingly, the relationship between MP abundance and particle sizes (sand, silt, and clay) weakens substantially during the post-monsoon season. A statistically weak correlation is seen between the MP concentration and sand (*r* = 0.01, *p* = 0.98, *n* = 9) (Table 3), and an insignificant negative correlation with silt (*r* =  − 0.20, *p* = 0.61) and clay fractions (*r* =  − 0.13, *p* = 0.74; Table 3). The weakening of the relationship is possibly due to the re-entrainment of sedimentary MPs due to extremely high rainfall in the region during July–August 2019 (Supplementary Figures S1–S3). Once the particles are re-entrained, they become buoyant and float until the river enters the Arabian Sea. They may finally sink and deposit with the marine sediments or be ingested by the marine organisms.

**Table 2:**
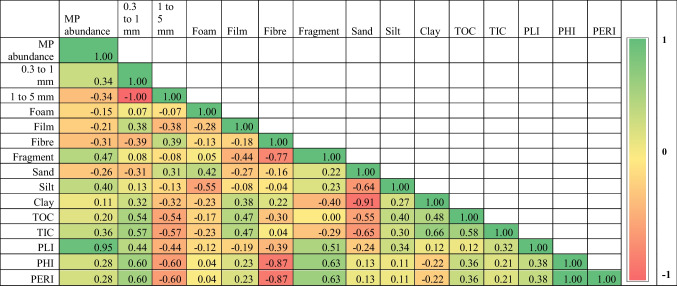
Correlation coefficient matrix for microplastics, sedimentology, organic, inorganic carbon, and risk assessment indices for sediments of River Sharavathi (pre-monsoon, *n* = 14)

**Table 3:**
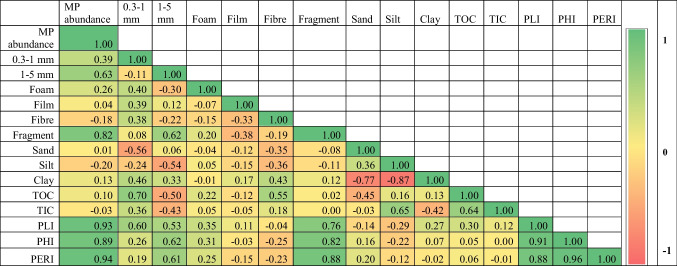
Correlation coefficient matrix for microplastics, sedimentology, organic, inorganic carbon, and risk assessment indices for sediments of River Sharavathi (post-monsoon, *n* = 9)

In summary, we observed an indifferent relationship between sediment particle size data and the abundance of MPs. The absence of a significant relationship can be attributed to various reasons. First, the difference in the physical processes acting on MPs and their transport mode can affect their behaviour in the sediments (Harris 2020). According to Peng et al. (2017), the complexity of hydrological conditions can be the reason for the lack of a relationship between particle size data and MP distribution. The physical properties of microplastics, including their size and density, can influence the relationship between the variables (Harris 2020). In addition, correlations between these variables would exist when MPs are large and dense enough to be transported by bedload and are available to be hydraulically sorted along with sediment grains (Harris 2020). Low-density microplastics with a larger surface area will only settle in the sediments due to specific processes, including ingestion, accumulation of pollutants, or biofouling (Falahudin et al. 2020). The transport of such particles is influenced by the mechanisms mentioned earlier rather than sedimentation processes. Here, microplastic particles and sediments are transported to the exact location by different processes, and therefore, it is not expected to find a relationship between them.

### Risk assessment of microplastics in sediments of River Sharavathi

The ubiquitous distribution of microplastics is a severe ecological problem, and it is associated with various harmful effects. Therefore, it is crucial to assess the risk posed by these hazardous materials, which will give a clear picture of the dangers they pose to the biota and their ecosystems located in heritage sites (Zhang et al. 2020; Adam et al. 2019). This will also help policymakers take the necessary steps to regulate plastic waste (Mitrano and Wohlleben 2020). In the present study, the PLI value of sediments ranges from 1 to 4.80 during PRM and from 0 to 2.45 during POM. The PLI scores for all the samples lie below 5, indicating that these samples belong to hazard level I (minor risk category), comparable to the average PLI scores reported for the Indian coast (5.58; Ranjani et al. 2021). The PLI is dependent on microplastic abundance and independent of chemical composition (Pan et al. 2020). This is evidenced by a highly positive correlation between PLI and MP abundance during both seasons (PRM: *r* = 0.98, *p* < 0.0001, *n* = 14; POM: *r* = 0.96, *p* < 0.0001, *n* = 9; Table 2). An increase in the PLI values (which is reflective of a higher MP abundance) points to significant anthropogenic activities in the region. Even though most of our study area lay in the densely forested Western Ghats, the region has witnessed a rise in human population and associated construction activities in the past decade (Ramachandra et al. 2012). According to Ranjani et al. (2021), sediments along the western coast of India are moderately polluted (hazard level II), derived from the PLI scores for selected regions (Maharashtra: 15.5; Karnataka:11.4; and Kerala (Vembanad Lake): 10.45). Furthermore, PLI scores of sediments from the eastern coast of India were reported to be less than 10 (hazard level I). Interestingly, the values of PLI obtained in the present study are lower than those documented from coastal Karnataka (Ranjani et al. 2021). The lower values may be attributed to the lower population density along the river's stretch than along the coastal stretch. The coastal regions are a hub for various anthropogenic activities (tourism, urbanisation, etc.), and, hence it is natural to obtain a higher PLI value.

**Fig. 6:**
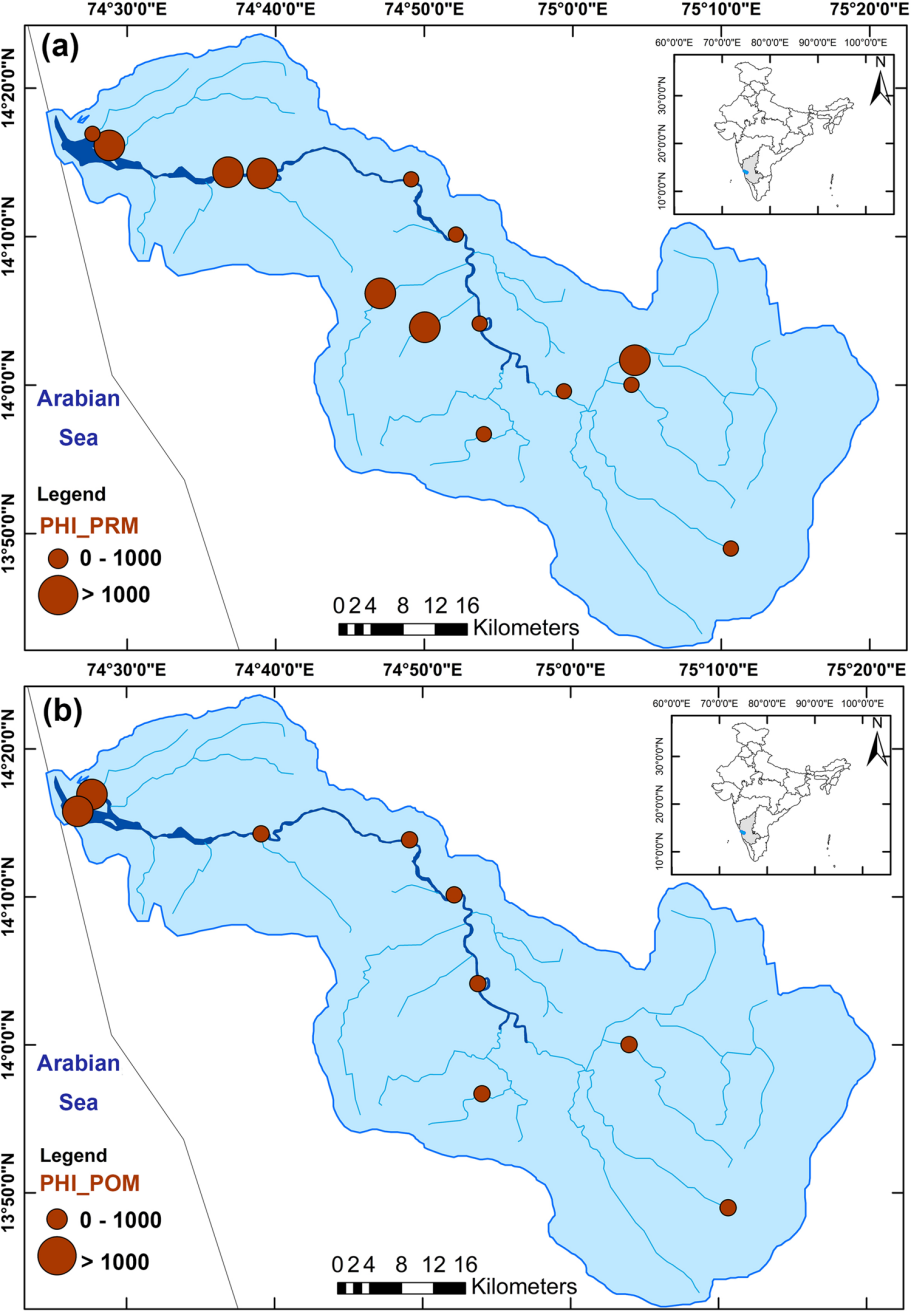
Polymer hazard index (PHI) along the River Sharavathi during **a** April 2019 (pre-monsoon), and **b** January 2020 (post-monsoon)

Based on the PHI values, Ranjani et al. (2021) found that the sediments in the coastal regions showed a serious microplastic pollution trend. The overall risk of microplastic pollution in India ranges from hazard level IV to V. The coastal areas of Karnataka have PHI values ranging from 100 to 1000, falling into hazard level IV. The present study’s PHI values vary between 400 and 1100 (pre-monsoon) and 0 to 1100 (post-monsoon; Fig. 6a and b). The risk of microplastic pollution can be classified as hazard level III to V (high to hazardous risk category). The higher PHI value is mainly due to an increased abundance of polyethylene; whose hazard score is more significant (11; Xu et al. 2018). Even in the absence of polymers such as polyvinyl chloride, polyamide, and polystyrene with high hazard scores, the risk factor is higher in the study area.

**Fig. 7:**
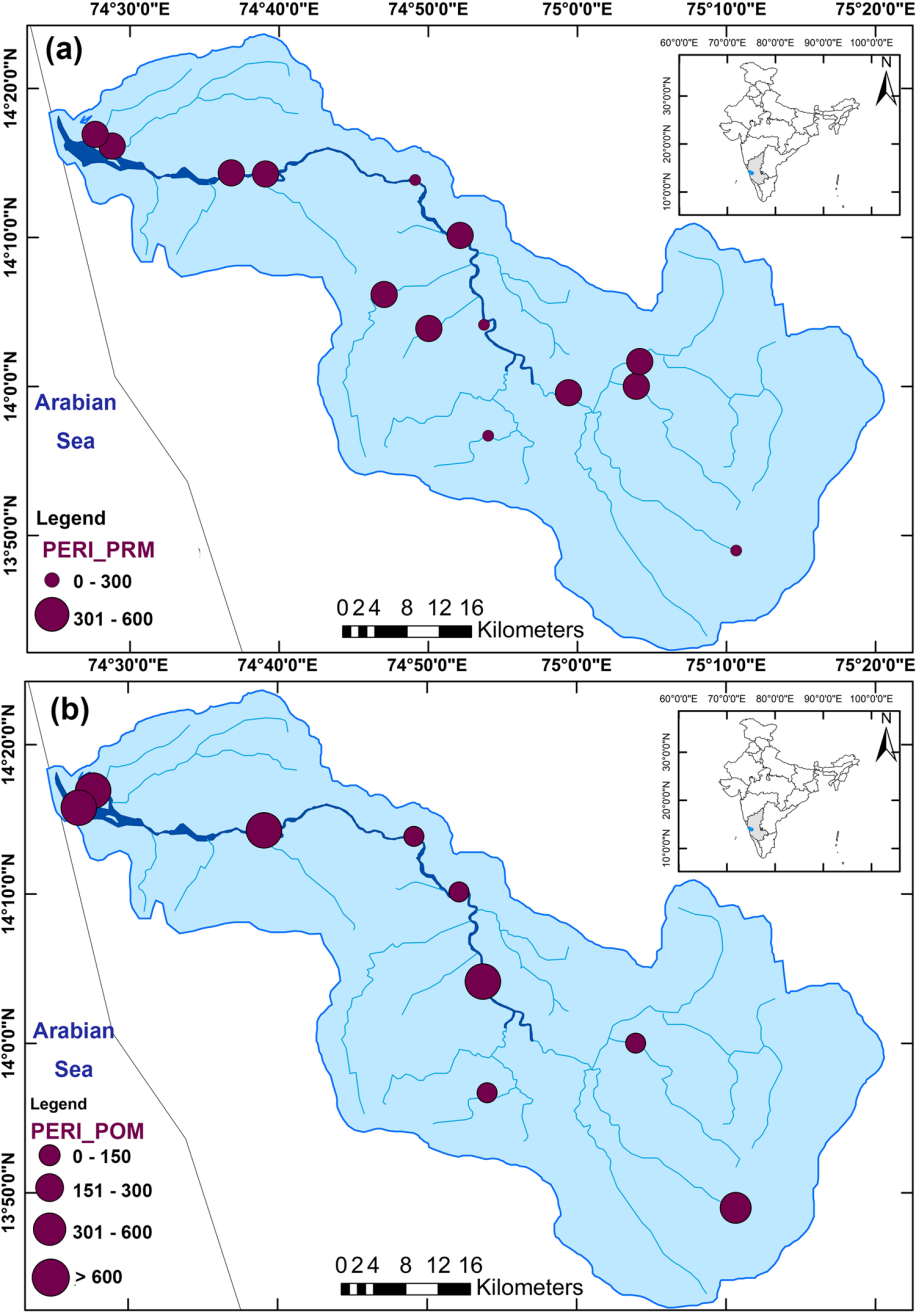
Potential ecological risk index (PERI) along the River Sharavathi during **a** April 2019 (pre-monsoon), and **b** January 2020 (post-monsoon)

The PERI scores (300 to 600) for sediments (continental and marine) along the Indian coast show the potential for high ecological risk, with an average PERI score of 303.2 for Karnataka (Ranjani et al. 2021). The present study’s PERI value ranges from 160 to 440 and 40 to 2240 during the PRM and POM (Fig. 7). In summary, the results indicate the significance of assessing chemical toxicity along with the microplastic concentration data. Risk assessment helps understand the extent of microplastic pollution in the region and its potential harm to human health (Xu et al. 2018). Even though the MP abundance is low, microplastic pollution in the study area poses a significant threat to the ecosystem, as shown by the risk factors (PHI and PERI). Besides, the risk contributed by 0.3–1-mm-sized microplastics is more significant as it offers a solidly positive relationship with the three indices and TOC % during the pre-monsoon (Table 2). However, the ties decrease during the post-monsoon season (Table 2), which could be due to the flushing out of microplastics during the extremely strong southwest monsoonal experienced by the region from June to September 2019.

## Implications of this study

The River Sharavathi is one of the pristine rivers originating in the Western Ghats region and hosts diverse organisms. Previous studies have shown the impacts of anthropogenic activities (hydroelectric projects) on the diversity of fish species like finfish (Bhat et al. 2014) and bivalve clams (Boominathan et al. 2014) in the estuarine region of the River Sharavathi. In this study, microplastics have been found in the sediments collected during the two seasons. Furthermore, a positive correlation is observed between films and TOC (*r* = 0.47, *p* = 0.07) and TIC (*r* = 0.47, *p* = 0.07) percentages during the pre-monsoon (Table 2). Similarly, a positive relationship is observed between TOC and fibres during the post-monsoon (*r* = 0.55, *p* = 0.09). Fibres are also positively correlated with clay fraction (*r* = 0.43, *p* = 0.21). The positive relationship indicates that the organic matter in the clay fraction is mostly adsorbed on the fibrous MPs. An earlier study by Maes et al. (2017) also observed a higher abundance of small-sized MPs in regions with high TOC. Many MPs were observed in estuarine areas whose sediments were organic-rich. The similarities in densities of MPs and sediment grains and the resulting sedimentation processes can explain this correlation (Maes et al. 2017). Therefore, areas with high total organic carbon content can be MP hotspots. Maes et al. (2017) inferred that, compared to benthic organisms in areas with larger sediment particle size and lower TOC, organisms burrowing and feeding in mud-rich environments are more susceptible to risks due to higher concentrations of microplastics. They also concluded that fine mud areas could be considered an essential hotspot for microplastics. In addition, particle size and organic matter can influence the metal bioavailability of organisms ingesting sediments. Furthermore, the increased surface area to volume ratio of MPs favours the accumulation of pollutants (Kazmiruk et al. 2018). Therefore, understanding the hotspots plays a vital role in regulating plastic pollution and its impact on the ecosystem. The evaluation of the three indices suggests that the usage of plastic materials in the catchment of the River Sharavathi has gradually increased over the years. As there is no proper solid waste management practise from source to sink in the region, the larger plastic pieces disintegrate into microplastics that remain in the sediments for a long time. Hence, MP researchers must focus on other pristine rivers in India and other countries to see any significant uptake of microplastics by flora and fauna in these aquatic systems.

## Conclusions

We studied the sediments of the River Sharavathi, *a pristine river* in the Western Ghats, a heritage site in southern India, to check the presence of MPs of various sizes and categories during the pre-monsoon and post-monsoon seasons. Besides, we analysed the textural properties and total organic and inorganic carbon of these sediments and explored their relationship with microplastics. The microplastic contamination in the sediments of the River Sharavathi is relatively low in concentration, with their abundance ranging from 2.5 to 57.5 pieces/kg (mean ± SD = 14.82 ± 3.10) during the pre-monsoon and 0 to 15 pieces/kg (mean ± SD = 6.39 ± 5.70) during the post-monsoon. The substantial dip in MPs’ abundance during the post-monsoon season is due to the extremely high rainfall in the river basin during July–August 2019, mainly due to MPs’ entrainment from the sediment and their subsequent transfer to the coastal region or the Arabian Sea. Smaller particles (0.3–1 mm) were more abundant than the more significant fractions (1–5 mm) in the sediments, mainly due to the breakdown of plastic materials deposited in the sediments by various physical actions. During both seasons, fragments predominated in the sediment samples. The main polymers were polyethylene and polyethylene terephthalate, with a minor concentration of polypropylene. Transparent and white-coloured MPs were the most abundant in the sediments.

No significant relationship was observed between the textural properties of sediments and microplastics, which may be due to the different behavioural properties of sediments and microplastics and particle shape and density. A good relationship was observed between small-sized MPs and organic and inorganic carbon. Larger (1–5 mm) and smaller (0.3–1 mm) MPs are negatively and positively correlated with TOC % and TIC %, respectively. The PLI, PHI, and PERI indices suggest different contamination levels in the river basin, and the district administration can use the data to mitigate plastic pollution. The study observed that microplastic distribution in the sediments of the river catchment is influenced by population distribution and their activities (land use and land cover), geomorphological features, hydrodynamic parameters, and processes such as precipitation, surface runoff, and sedimentation.

The study provides a baseline for the spatial distribution of microplastics which will be helpful in understanding the hotspot of microplastic pollution in the River Sharavathi catchment. The data can be used by policymakers to take initiatives to minimise pollution. In order to curb the global problem of marine plastic pollution, the movement of plastic materials from the rivers to the marine environment should be checked. In addition to the five Rs—refuse, reduce, reuse, recycle, and recover—more initiatives must be taken to establish advanced waste management techniques. Also, new initiatives should be taken to restrict the flow of plastic waste from aquatic systems such as rivers and lakes to seas and oceans. One such example is the employment of trash booms to collect floating plastic materials from the rivers before they enter the marine system (Plastic Fischer 2022). As the area is recognised as a biodiversity hotspot, along with the concentration data, we tried to find out the risk posed by microplastics obtained from the catchment. Knowledge of these factors can be used by policymakers and environmental managers to investigate the issue and take regulatory action.

## Supplementary Information

Below is the link to the electronic supplementary material.

Supplementary file1 (DOCX 626 KB)

Supplementary file2 (DOC 63 KB)

## Data Availability

Data will be made available upon request.
