# Supplementary material for: Assessment of pollution and risks associated with microplastics in the riverine sediments of the Western Ghats: a heritage site in southern India
Source: Environ Sci Pollut Res Int. 2022 Dec 3;30(12):32301–19. doi: 10.1007/s11356-022-24437-z (PMC10017654; doi:10.1007/s11356-022-24437-z)
Supplement: Supplementary file 1 — Supplementary file1 (DOCX 626 KB) [file 11356_2022_24437_MOESM1_ESM.docx]

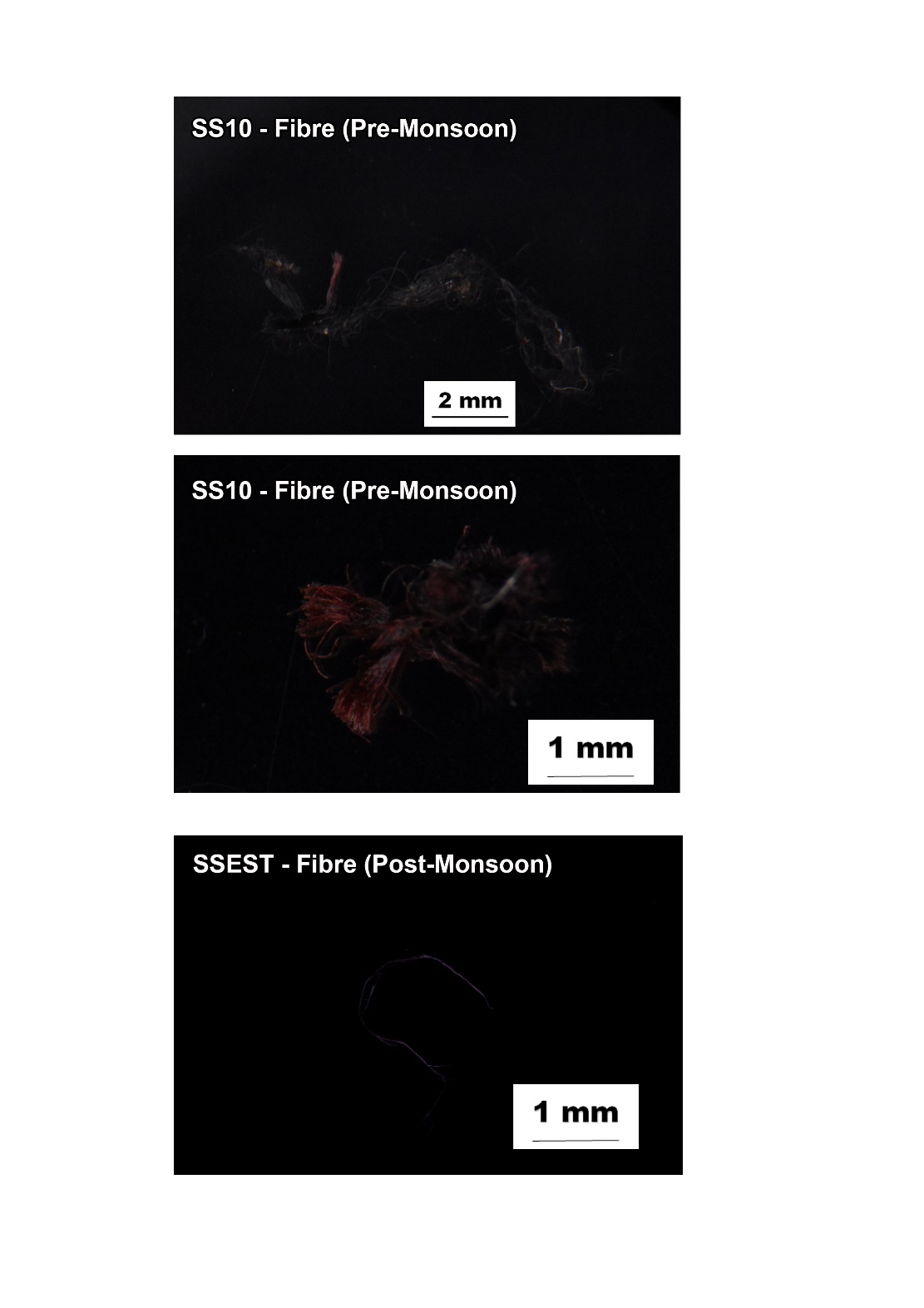


**Fig. S1.**  Microphotographs of the fibres found in this study. Scale bar is in millimetre.

**
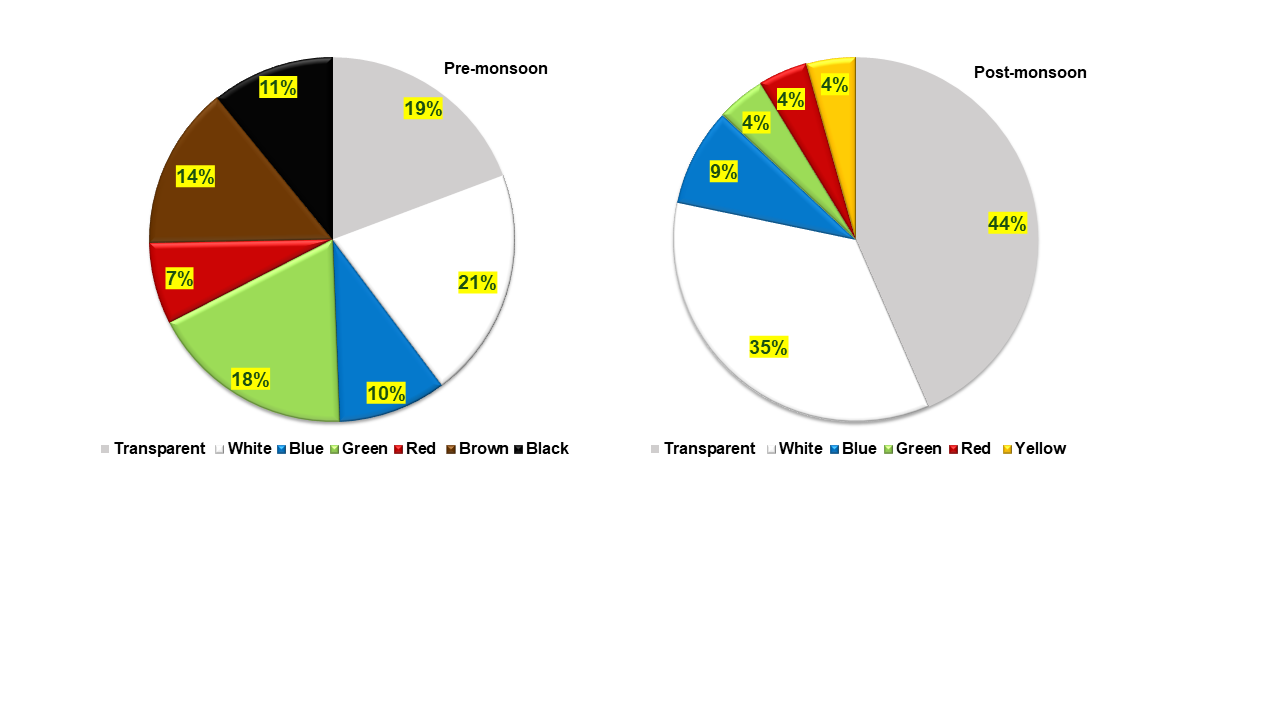
**

**Fig. S2.** Pie charts showing the abundance of different colours of microplastics present in sediment samples of the River Sharavathi.

**
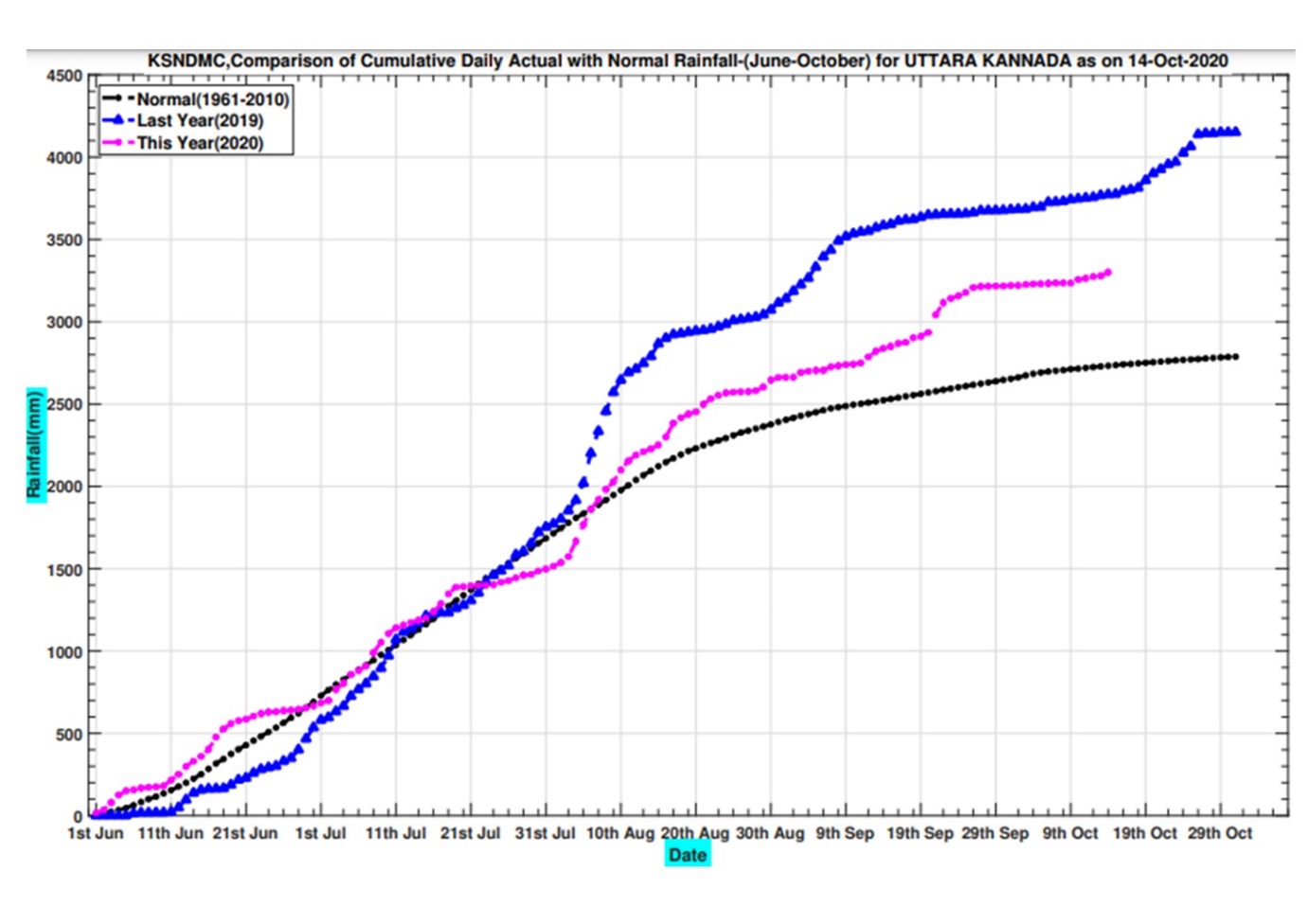
**

**Fig. S3.** Daily rainfall data of the 2019 and 2020 monsoon seasons for the Uttara Kannada District through which River Sharavathi flows (Karnataka State Natural Disaster Monitoring Cell, 2020). Note the significantly higher southwest monsoonal rainfall received in the region during the June to October 2019 (blue curve). The samples for this study were collected during May 2019 (pre-monsoon) and January 2020 (post-monsoon).

**
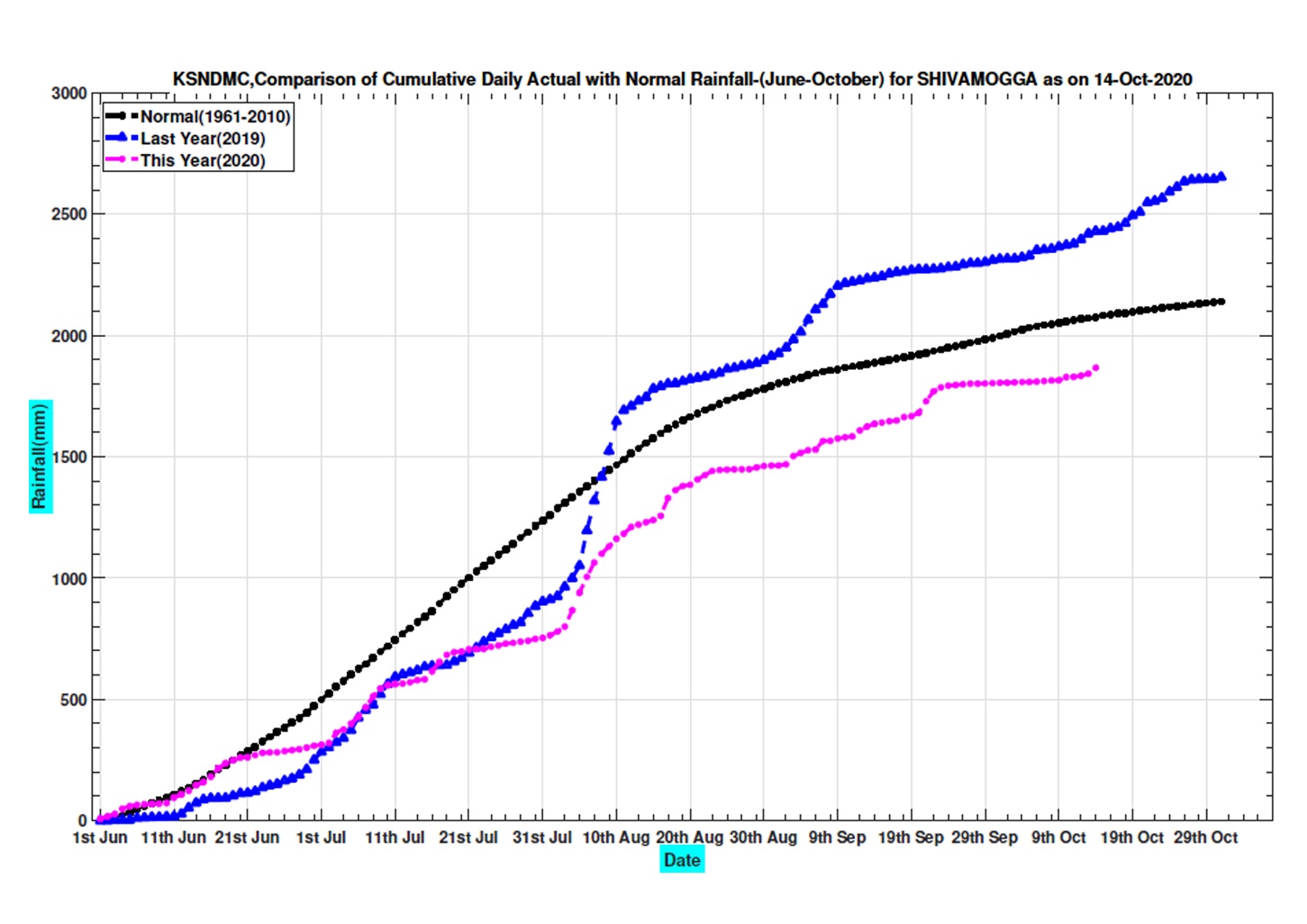
**

**Fig. S4.** Daily rainfall data of the 2019 and 2020 monsoon seasons for the Shivamogga District through which River Sharavathi flows (Karnataka State Natural Disaster Monitoring Cell, 2020). Note the significantly higher southwest monsoonal rainfall received in the region during the June to October 2019 (blue curve). The samples for this study were collected during May 2019 (pre-monsoon) and January 2020 (post-monsoon).
