# Supplementary material for: Assessment of pollution and risks associated with microplastics in the riverine sediments of the Western Ghats: a heritage site in southern India
Source: Environ Sci Pollut Res Int. 2022 Dec 3;30(12):32301–19. doi: 10.1007/s11356-022-24437-z (PMC10017654; doi:10.1007/s11356-022-24437-z)
Supplement: Supplementary file 2 — Supplementary file2 (DOC 63 KB) [file 11356_2022_24437_MOESM2_ESM.doc]

**Supplementary Table S1.** Sedimentological, total organic and inorganic carbon data for River Sharavathi sediments collected during the pre-monsoon (n=14) and post-monsoon (n=9). The statistical summary of the data is also presented; SD is standard deviation.

| **Sl. No.** | **Sample ID** | **Sample Location** | **Sand (%)** | **Silt (%)** | **Clay (%)** | **TOC (%)** | **TIC (%)** | **Season** |
| --- | --- | --- | --- | --- | --- | --- | --- | --- |
| 1 | SS1 | Achakanya | 80.31 | 13.12 | 6.56 | 0.09 | 0.03 | Pre-monsoon (May, 2019) |
| 2 | SS2 | Sidyapura | 30.00 | 26.69 | 43.31 | 0.35 | 0.10 |  |
| 3 | SS3 | Tottadikappe | 48.28 | 17.47 | 34.25 | 0.33 | 0.05 |  |
| 4 | SS4 | Mavinahole | 42.09 | 17.72 | 40.19 | 0.33 | 0.10 |  |
| 5 | SS5 | Tonikale | 15.68 | 13.88 | 70.44 | 0.73 | 0.13 |  |
| 6 | SS6 | Linganamakki | 16.52 | 24.46 | 59.02 | 0.60 | 0.10 |  |
| 7 | SS7 | Valagere | 8.46 | 31.52 | 60.02 | 2.18 | 0.14 |  |
| 8 | SS8 | Kalur | 42.69 | 23.41 | 33.90 | 0.69 | 0.16 |  |
| 9 | SS9 | Talagoppe | 20.37 | 42.23 | 37.40 | 0.77 | 0.09 |  |
| 10 | SS10 | Jog Falls | 27.16 | 33.62 | 39.22 | 0.36 | 0.06 |  |
| 11 | SS11 | Gersoppa Ferry | 59.65 | 24.72 | 15.64 | 0.77 | 0.06 |  |
| 12 | SS12 | Samshi | 92.20 | 5.20 | 2.60 | 0.28 | 0.03 |  |
| 13 | SS13 | Hosadmane | 36.34 | 34.92 | 28.74 | 0.81 | 0.14 |  |
| 14 | SS14 | Besinekere | 50.31 | 9.32 | 40.37 | 0.70 | 0.11 |  |
| ***Statistical summary*** | | ***Average*** | **40.72** | **22.73** | **36.55** | ***0.64*** | ***0.09*** |  |
|  |  | ***Maximum*** | **92.20** | **42.23** | **70.44** | ***2.18*** | ***0.16*** |  |
|  |  | ***Minimum*** | **8.46** | **5.20** | **2.60** | ***0.09*** | ***0.03*** |  |
|  |  | ***SD*** | **24.36** | **10.59** | **19.31** | ***0.50*** | ***0.04*** |  |
| 1 | SS1 | Achakanya | 29.93 | 5.50 | 64.58 | 0.10 | 0.02 | Post-monsoon (2019-2020) |
| 2 | SS2 | Sidyapura | 30.84 | 28.44 | 40.72 | 0.33 | 0.03 |  |
| 3 | SS5 | Tonikale | 15.16 | 40.58 | 44.27 | 0.54 | 0.09 |  |
| 4 | SS6 | Linganamakki | 28.35 | 30.06 | 41.59 | 0.50 | 0.07 |  |
| 5 | SS9 | Talagoppe | 18.98 | 15.98 | 65.04 | 0.65 | 0.08 |  |
| 6 | SS10 | Jog Falls | 45.52 | 51.97 | 2.51 | 0.26 | 0.10 |  |
| 7 | SS11 | Gersoppa Ferry | 35.06 | 25.68 | 39.26 | 0.33 | 0.05 |  |
| 8 | SS14 | Besinekere | 42.52 | 31.69 | 25.80 | 0.50 | 0.09 |  |
| 9 | SSEST | Sharavathi Estuary | 19.79 | 23.65 | 56.56h | 0.39 | 0.08 |  |
| ***Statistical summary*** | | ***Average*** | **29.57** | **28.17** | **42.26** | ***0.40*** | ***0.07*** |  |
|  |  | ***Maximum*** | **45.52** | **51.97** | **65.04** | ***0.65*** | ***0.10*** |  |
|  |  | ***Minimum*** | **15.16** | **5.50** | **2.51** | ***0.10*** | ***0.02*** |  |
|  |  | ***SD*** | **10.434** | **13.35** | **19.647** | ***0.17*** | ***0.03*** |  |
